# Green tea intake is associated with urinary estrogen profiles in Japanese-American women

**DOI:** 10.1186/1475-2891-12-25

**Published:** 2013-02-15

**Authors:** Barbara J Fuhrman, Ruth M Pfeiffer, Anna H Wu, Xia Xu, Larry K Keefer, Timothy D Veenstra, Regina G Ziegler

**Affiliations:** 1 Division of Cancer Epidemiology and Genetics, National Cancer Institute, National Institutes of Health, Bethesda, MD, USA; 2 SAIC-Frederick, Inc, National Cancer Institute at Frederick, Frederick, MD, USA; 3 University of Southern California Keck School of Medicine, Los Angeles, CA, USA; 4 National Cancer Institute at Frederick, Frederick, MD, USA; 5 Current address: Department of Epidemiology, Fay W. Boozman College of Public Health, University of Arkansas for Medical Sciences, 4301 W. Markham St. #820, Little Rock, AR, 72205, USA

**Keywords:** Estrogens, Metabolism, Green tea, Camellia sinensis, Breast neoplasms, Risk factors, Human, Female, Middle-aged

## Abstract

**Scope:**

Intake of green tea may reduce the risk of breast cancer; polyphenols in this drink can influence enzymes that metabolize estrogens, known causal factors in breast cancer etiology.

**Methods and results:**

We examined the associations of green tea intake (<1 time/week, 1-6 times weekly, or 7+ times weekly) with urinary estrogens and estrogen metabolites (jointly EM) in a cross-sectional sample of healthy Japanese American women, including 119 premenopausal women in luteal phase and 72 postmenopausal women. We fit robust regression models to each log-transformed EM concentration (picomoles per mg creatinine), adjusting for age and study center. In premenopausal women, intake of green tea was associated with lower luteal total EM (P trend = 0.01) and lower urinary 16-pathway EM (P trend = 0.01). In postmenopausal women, urinary estrone and estradiol were approximately 20% and 40% lower (P trend = 0.01 and 0.05, respectively) in women drinking green tea daily compared to those drinking <1 time/week. Adjustment for potential confounders (age at menarche, parity/age at first birth, body mass index, Asian birthplace, soy) did not change these associations.

**Conclusions:**

Findings suggest that intake of green tea may modify estrogen metabolism or conjugation and in this way may influence breast cancer risk.

## Background

Breast cancer is the leading cancer diagnosis among women worldwide. There is great variability in international breast cancer incidence rates. The incidence of breast cancer is three to four times higher in the U.S. compared to Japan [1]. Migrant studies suggest that international differences in breast cancer risk are mediated by environmental rather than genetic factors [2].

Green tea is commonly consumed in Japan. Green tea is an important source of dietary phytochemicals, including the polyphenols epigallocatechin gallate (EGCG), epigallocatechin, epicatechin gallate, and epicatechin. Together these phytochemicals represent 30% of the dry weight of fresh tea leaves [3]. Green tea polyphenols inhibit tumor development in laboratory models of breast cancer and other cancers, but epidemiologic evidence does not consistently support the hypothesis that green tea drinking lowers breast cancer risk [4]. Mechanisms of anticancer actions may include interruption of signal transduction pathways involved in cell proliferation, invasion, angiogenesis, and metastasis [3,5].

Because green tea catechins have been observed to reduce catalytic activity of certain cytochrome p450 (CYP) enzymes [6], we hypothesized that green tea may influence breast cancer risk, in part, by modifying the production or metabolism of estrogens, known carcinogens of the breast. Caffeine, present in teas and other foods, is also known to modify expression and/or activity of some metabolic enzymes, but has not been found to have a consistent effect on breast cancer risk [7-10].

Therefore we examined the associations of green tea intake and caffeine intake with urinary estrogens and estrogen metabolites (jointly referred to as EM) among cancer-free pre- and postmenopausal Japanese American women.

## Methods

### Study population

Subjects for this analysis were Japanese-American control subjects who participated in the population-based Asian American Breast Cancer case-control study that has already been described in detail [2]. In that study, cases were women of Chinese, Japanese or Filipino descent, aged 20-55 years, diagnosed with histologically-confirmed, first primary breast cancers in San Francisco-Oakland, California; Los Angeles, California; or Oahu, Hawaii, between April 1, 1983 and June 30, 1987. Potential controls in San Francisco and Los Angeles were selected by random-digit dialing, while those in Oahu were selected from an annual population-based health survey conducted by the Health Surveillance Program of the Hawaii Department of Health. Bilingual interviewers were used when necessary. Controls, up to two per case, were frequency-matched to cases in their study area by Asian ethnicity and year of birth in 5-year age groups.

Study participants were interviewed at home by trained staff with standardized questionnaires, which included questions about residential history, birthplaces of parents and grandparents, medical history, and dietary and lifestyle factors. Only Japanese American participants were queried about intake of green tea. Participants were also invited to provide 12-hour overnight urine and/or fasting blood samples. Women who reported a recent menstrual period were given urine collection appointments to coincide with the mid-luteal phase (days 19-26) of the menstrual cycle and were asked to send a postcard following urine collection to report the first day of the subsequent menstrual period.

### Ethical approval

All participants provided informed consent. The research was approved by the responsible Institutional Review Board at the National Cancer Institute.

### Menopausal status

We assessed menopausal status carefully using data from multiple sources; decision rules were designed to identify women who were clearly post-menopausal and those who were premenopausal and in luteal phase. Postmenopausal women included women who had experienced natural menopause at least 12 months earlier. Other women without evidence of ongoing menses at the time of urine collection and those who had reported surgical menopause were considered postmenopausal only if they had circulating FSH ≥ 35 U/L and either progesterone < 10 ng/dL, progesterone < 30 ng/dL and estradiol < 35 ng/dL, or progesterone < 50 ng/dL and estradiol < 15 ng/dL. For those women missing the FSH reading, postmenopausal status was assigned to those aged >52 y at the time of urine collection and who had progesterone < 30 ng/dL and estradiol < 35 ng/dL or progesterone < 50 ng/dL and estradiol < 15 ng/dL. For premenopausal women, phase of cycle was based on 1) the returned postcards and 2) serum progesterone levels. A premenopausal woman was considered to be in luteal phase if her urine collection fell 2–11 days before the subsequent menstrual period or if her progesterone levels were >300 ng/dL.

### Inclusion / exclusion criteria

Eligible participants in this study were Japanese-American women without breast cancer (n = 395). Of these, 284 (72%) provided 12-hour urine samples. We then excluded participants who reported at the time of urine collection current or recent use (<6 months) of exogenous hormones (n = 45), or current / recent (<6 months) pregnancies or lactation (n = 5), resulting in a sample of 234 women. Of these, 119 were classified as premenopausal and in luteal phase at the time of urine collection, 72 were classified as postmenopausal, and 43 were either premenopausal but not in luteal phase, or of uncertain menopausal status. The latter group of 43 women was excluded to avoid variability by menstrual phase [11]. Therefore the present study included 119 premenopausal women in luteal phase and 72 postmenopausal women.

### Specimen handling and assay for urinary estrogens and estrogen metabolites

Participants were instructed to collect 12-hour overnight urine samples using half-gallon containers kept at 4°C on ice or in the refrigerator. Boric acid was added as a preservative [12]. After delivery to the study center, urine was mixed, aliquoted into 1 mL cryovials, and shipped on dry ice to a repository for storage at -80°C.

Unthawed aliquots were sent to SAIC-Frederick (Frederick, MD) where stable isotope dilution high-performance liquid chromatography-tandem mass spectrometry (LC/MS/MS) was used to measure concurrently 15 urinary estrogens and estrogen metabolites, including estrone, estradiol, estriol, 2-hydroxyestrone, 2-methoxyestrone, 2-hydroxyestradiol, 2-methoxyestradiol, 2-hydroxyestrone-3-methyl ether), 4-hydroxyestrone, 4-methoxyestrone, 4-methoxyestradiol, 16α-hydroxyestrone, 17-epiestriol 16-ketoestradiol, and 16-epiestriol. The analytical method for measurement of urinary estrogens and estrogen metabolites has been described previously [13], as have the assay conditions as applied to this particular study [14].

Urine samples from two premenopausal and two postmenopausal women were used as blinded quality control samples. Four quality control samples, including two from the same subject, were randomly included in each batch of ~40 samples. Total laboratory coefficients of variation were <10% for all estrogens and estrogen metabolites except 4-methoxyestradiol (15%), and were ≤4% for estrone, estradiol, and estriol, and ~1% for total EM.

Creatinine levels were determined by the National Institutes of Health clinical chemistry laboratory and used to adjust EM levels for differences in urine concentration [15].

### Tea and other dietary measures

During in-person interviews, Japanese-American participants were queried about frequency of intake of green tea, black tea, coffee, decaffeinated coffee and caffeinated soda; serving size was not queried. Frequencies of intake were recorded as times per day, week, month, or year, according to the most convenient time frame for the food item and respondent. Based on observed distributions for included participants, green tea consumption frequency was classified as less than once per week, weekly (1-6 servings per week), or daily (7 or more servings per week). Caffeine was calculated assuming standard serving sizes and the following estimates of caffeine content, based on food composition databases [16] and measures in the literature [17]: 81 mg caffeine per serving of coffee, 2 mg caffeine per serving of decaffeinated coffee, 20 mg caffeine per serving of green tea, 30 mg per serving of black tea, and 29 mg per serving of caffeinated soda.

### Statistics and data analysis

We examined characteristics of study participants in premenopausal women who provided urine during the luteal phase of the menstrual cycle, and in postmenopausal women (Table 1) and by category of tea intake (Table 2). Estrogens and estrogen metabolites were grouped by metabolic pathway, summed and expressed in picomoles per mg creatinine. We have previously shown that these pathway categories appropriately characterize covariation in urinary EM profiles [14].

**Table 1:** Characteristics of breast cancer controls in the Asian-American Breast Cancer Study by menopausal status

	**Premenopausal women in luteal phase (n = 119)**		**Postmenopausal women (n = 72)**	
	**mean**	**s.d.**	**mean**	**s.d.**
Age (y)*	41.2	5.4	53.0	3.4
**Body Mass Index (kg / m**^**2**^**)**	21.7	3.5	21.7	2.5
	**n**	**column %**	**n**	**column %**
**Study center, n(%)**				
Hawaii	79	66.4%	48	66.7%
Los Angeles	19	16.0%	13	18.1%
San Francisco	21	17.6%	11	15.3%
**Combined parity and age at first birth, n(%)**				
Nulliparous	17	14.3%	11	15.3%
1st birth age ≤ 20 y	7	5.9%	4	5.6%
1-2 live births / age ≥ 21	73	61.3%	28	38.9%
3+ live births / age ≥ 21	22	18.5%	29	40.3%
**Age at menarche, n(%)**				
≤ 12 y	72	60.5%	30	41.7%
> 12 y	47	39.5%	42	58.3%
**Born in Asia, n (%)**				
No	93	78.2%	57	79.2%
Yes	26	21.8%	15	20.8%
**Soy intake (frequency of weekly intake), n (%)**				
0-0.5	35	29.4%	12	16.7%
0.5 – 1.0	35	29.4%	22	30.6%
> 1.0	49	41.2%	38	52.8%
**Green tea (frequency of weekly intake), n (%)**				
<once weekly	62	52.1%	25	34.7%
1-6 times weekly	32	26.9%	23	31.9%
7+ times weekly	25	21.0%	24	33.3%
**Black tea (frequency of weekly intake), n (%)**				
<once weekly	85	71.4%	51	70.8%
1-6 times weekly	21	17.6%	16	22.2%
7+ times weekly	13	10.9%	5	6.9%
**Coffee (frequency of weekly intake), n (%)**				
0-2 times weekly	30	25.2%	11	15.3%
3-13 times weekly	39	32.8%	22	30.6%
≥14 times weekly	50	42.0%	39	54.2%
**Estimated weekly caffeine intake (mg), n (%)**				
0-734	41	34.5%	23	31.9%
734-1610	39	32.8%	23	31.9%
>1610	39	32.8%	26	36.1%

**Table 2:** Participant characteristics by frequency of green tea intake

	**Less than weekly intake (< once per week)**		**Weekly intake (1-6 times per week)**		**Daily intake (7+ times per week)**		P value*
	**mean**	**s.d.**	**mean**	**s.d.**	**mean**	**s.d.**	
Age (y)*	45.4	6.8	48.3	8.2	49.7	7.0	0.01
**Body Mass Index (kg / m**^**2**^**)**	21.9	3.3	22.0	3.1	21.0	2.8	0.18
	**n**	**row %**	**n**	**row %**	**n**	**row %**	
**Menopausal status / menstrual phase**							0.049
Premenopausal women in luteal phase	62	52.1%	32	26.9%	25	21.0%	
Postmenopausal	25	34.7%	23	31.9%	24	33.3%	
**Study center, n (%)**							0.01
Hawaii	70	55.1%	34	26.8%	23	18.1%	
Los Angeles	7	21.9%	13	40.6%	12	37.5%	
San Francisco	10	31.3%	8	25.0%	14	43.8%	
**Combined parity and age at first birth, n (%)**							0.07
Nulliparous	12	42.9%	8	28.6%	8	28.6%	
1st birth age ≤ 20 y	7	63.6%	4	36.4%	0	.0%	
1-2 live births / age ≥ 21	53	52.5%	23	22.8%	25	24.8%	
3+ live births / age ≥ 21	15	29.4%	20	39.2%	16	31.4%	
**Age at menarche, n (%)**							<0.001
≥ 12 y	56	54.9%	34	33.3%	12	11.8%	
> 12 y	31	34.8%	21	23.6%	37	41.6%	
**Born in Asia, n (%)**							<0.001
No	74	49.3%	49	32.7%	27	18.0%	
Yes	13	31.7%	6	14.6%	22	53.7%	
**Soy intake (frequency of weekly intake), n (%)**							<0.001
0-0.5 times weekly	31	66.0%	12	25.5%	4	8.5%	
0.5-1 times weekly	27	47.4%	20	35.1%	10	17.5%	
> once weekly	29	33.3%	23	26.4%	35	40.2%	
**Black tea (frequency of weekly intake), n (%)**							0.21
< once weekly	69	50.7%	34	25.0%	33	24.3%	
1-6 times weekly	12	32.4%	15	40.5%	10	27.0%	
7+ times weekly	6	33.3%	6	33.3%	6	33.3%	
**Coffee (frequency of weekly intake), n (%)**							0.75
0-2 times weekly	22	53.7%	11	26.8%	8	19.5%	
3-13 times weekly	25	41.0%	18	29.5%	18	29.5%	
≥14 times weekly	40	44.9%	26	29.2%	23	25.8%	
**Caffeine (weekly intake in mg), n (%)**							0.83
0-734	29	50.0%	15	25.9%	14	24.1%	
734-1609	27	47.4%	15	26.3%	15	26.3%	
≥1610	31	40.8%	25	32.9%	20	26.3%	

* For continuous covariates, p-values were obtained using ANOVA and reflect significance of differences between means by categories of green tea intake. For categorical covariates, p-values were obtained using Chi Square tests and reflect differences in distribution.

Direct standardization was used to calculate representative geometric means of EM measures for Japanese-American women by categories of green tea intake. Age standardization was performed with the log transforms of each EM measure, and the results were then exponentiated. For premenopausal women in luteal phase, standardization was to the age distribution of all premenopausal women in luteal phase (<40, 40-44, 45+ y), regardless of tea intake For postmenopausal women, standardization was to the age distribution of all postmenopausal women (<50, 50-54, 55+ y).

Robust regression models were fit to each log-transformed EM measure as the dependent variable. Regression coefficients associated with each of the tea categories in linear models were used to estimate percent differences based on the formula: 100*(E^beta^ – 1), where beta is the coefficient associated with each category of tea intake.

Models were fit separately for pre- and postmenopausal women and were adjusted for age and study center. We also considered additional measures as potential confounders including birthplace, soy intake, age at menarche, parity/ age at first birth, and body mass index (BMI). Models that additionally adjusted for caffeine intake were also considered. The statistically significant findings in Tables 3 and 4 did not differ in their direction, magnitude, or statistical significance when additional potential confounders were included in models. Here we present the minimally adjusted models.

**Table 3:** **Age standardized geometric means for urinary concentrations of estrogens and estrogen metabolites (EM) and multivariable-adjusted percent difference**^**1**^**across categories of green tea intake in premenopausal women during the luteal phase**

	**Categories of green tea intake**					
	**<1 per week (n = 62)**	**1-6 times per week (n = 32)**		**7+ times per week (n = 25)**		
	**Urinary concentration**	**Urinary concentration**	**Percent difference**	**Urinary concentration**	**Percent difference**	***P*trend**
**Total Estrogens and Metabolites**	218.16	156.13	**−25.5% (-40.7%-**^**-**^**6.4%)**	170.36	**−26.3% (-43.0%-**^**-**^**4.6%)**	**0.01**
**Parent Estrogens**	37.58	29.99	−16.0% (-35.2%-8.9%)	34.57	−8.1% (-31.7%-23.6%)	0.42
Estrone	24.45	19.44	−20.2% (-39.2%-4.8%)	23.07	−6.9% (-31.8%-27.2%)	0.43
Estradiol	11.53	10.01	−5.4% (-27.0%-22.5%)	10.28	−8.9% (-32.1%-22.3%)	0.51
**2-Hydroxylation pathway**	39.11	28.05	**−36.9% (-52.3%-**^**-**^**16.4%)**	38.11	−16.2%(-39.5%-16.0%)	0.10
**2-Pathway catechols**	30.57	22.42	**−38.3%(-54.2%-**^**-**^**16.8%)**	28.5	−16.9%(-41.2%-17.6%)	0.10
2-Hydroxyestrone	27.65	19.68	**−38.8%(-54.6%-**^**-**^**17.6%)**	27.06	−16.3%(-40.7%-18.2%)	0.10
2-Hydroxyestradiol	3.02	1.90	**−43.9%(-61.9%-**^**-**^**17.3%)**	2.61	−25.5% (-52.4%-16.5%)	0.07
**2-Pathway methylated catechols**	7.24	5.66	**−28.9%(-47.7%-**^**-**^**3.5%)**	7.77	−9.6%(-36.3%-28.2%)	0.34
2-Methoxyestrone	5.17	4.18	−24.0% (-44.6%-4.2%)	5.74	−1.3%(-31.4%-41.9%)	0.67
2-Methoxyestradiol	0.56	0.41	−28.7% (-50.8%-3.3%)	0.64	0.6% (-34.0%-53.4%)	0.73
2-Hydroxyestrone-3-methyl ether	1.15	0.81	**−34.1% (-56.3%-**^-^**0.5%)**	1.06	−22.9%(-51.5%-22.5%)	0.17
**4-Hydroxylation pathway EM**	4.13	3.32	−21.4% (-43.3%-8.9%)	3.80	−13.5% (-40.3%-25.3%)	0.33
4-Pathway catechol: 4-Hydroxyestrone	3.71	2.95	−21.9% (-44.3%-9.6%)	3.31	−15.5% (-42.4%-24.1%)	0.29
**4-Pathway methylated catechols**	0.33	0.28	−19.1% (-43.3%-15.3%)	0.36	−7.1% (-38.0%-39.2%)	0.53
4-Methoxyestrone	0.23	0.17	−23.9% (-48.1%-11.6%)	0.24	4.0% (-32.5%-60.4%)	0.93
4-Methoxyestradiol	0.05	0.06	−15.2% (-56.1%-63.6%)	0.08	−8.2% (-56.4%-93.4%)	0.77
**16-Hydroxylation pathway**	123.03	84.72	−21.3% (-39.7%-2.7%)	87.57	**−33.3% (-50.3% -**^**-**^**10.4%)**	**0.001**
16α-Hydroxyestrone	12.68	9.31	**−24.0%(-40.9%-**^**-**^**2.3%)**	9.78	**−31.1% (-48.2%-**^**-**^**8.5%)**	**0.01**
Estriol	68.67	44.67	−23.5% (-43.2%-2.9%)	46.16	**−33.1% (-51.9%-**^**-**^**7.0%)**	**0.01**
16-Ketoestradiol	25.68	19.98	−17.8% (-39.8%-12.2%)	19.32	**−31.5% (-51.3%-**^**-**^**3.7%)**	**0.02**
16-Epiestriol	10.09	7.11	−15.1% (-36.6%-13.6%)	7.74	**−30.8% (-49.9%-**^**-**^**4.2%)**	**0.02**
17-Epiestriol	1.79	0.97	−27.4% (-54.9%-16.8%)	0.95	**−40.4% (-65.1%-1.7%)**	**0.04**
**Ratios**						
**Parent estrogens / estrogen metabolites**	0.21	0.24	10.6% (-11.5%-38.3%)	0.26	23.1% (-3.8%-57.6%)	0.09
**2-Hydroxylation pathway / parent estrogens**	1.04	0.94	−19.3% (-37.3%-3.8%)	1.10	−9.5% (-31.7%-20.1%)	0.37
**4-Hydroxylation pathway / parent estrogens**	0.11	0.11	−9.9% (-32.9%-21.2%)	0.11	−15.8% (-39.2%-16.6%)	0.28
**16-Hydroxylation pathway / parent estrogens**	3.27	2.83	−5.1% (-27.8%-24.8%)	2.53	−20.0% (-41.0%-8.5%)	0.17
**2-Hydroxylation pathway / 16-hydroxylation pathway**	0.32	0.33	−3.7% (-33.2%-38.8%)	0.44	21.2% (-19.8%-83.0%)	0.41
**4-Hydroxylation pathway / 16-hydroxylation pathway**	0.03	0.04	16.8% (-21.3%-73.3%)	0.04	21.8% (-21.8%-89.8%)	0.34
**2-Hydroxylation pathway / 4-hydroxylation pathway**	9.47	8.46	−13.3% (-28.3%-4.7%)	10.04	−1.2% (-20.2%-22.4%)	0.70
**4-Pathway methylated catechols / 4-pathway catechols**	0.09	0.09	20.7% (-13.4%-68.1%)	0.11	23.9% (-15.2%-81.0%)	0.20
**2-Pathway methylated catechols / 2-pathway catechols**	0.23	0.26	9.1% (-11.6%-34.6%)	0.26	7.7% (-15.1%-36.7%)	0.48

Derived measures of estrogen metabolism and statistically significant estimates are presented in bold font.

^1^ Robust linear regression models were used to estimate percent difference in EM measures with 95% confidence limits across categories of green tea intake while adjusting for age and study center.

^2^ Robust linear regression was used to test for trends in log-transformed EM measures across tea categories. P_trend_ was modeled using categories coded as 0, 1, and 2.

**Table 4:** **Age-standardized mean urinary concentrations (picomoles per mg creatinine) of estrogens and estrogen metabolites (EM) and multivariable-adjusted percent difference**^**1**^**across categories of green tea intake in postmenopausal women**

	**Categories of green tea intake**					
	**<1 per week (n = 62)**	**1-6 times per week (n = 32)**		**7+ times per week (n = 25)**		
	**Urinary concentration**	**Urinary concentration**	**Percent difference**	**Urinary concentration**	**Percent difference**	***P*trend**
**Total Estrogens and Metabolites**	27.7	26.7	−6.2% (-33.7%-32.6%)	26.5	−10.5% (-36.0%-25.1%)	0.52
**Parent Estrogens**	5.81	3.89	**−37.0% (-58.0%-**^**-**^**5.4%)**	4.01	**−38.6% (-58.3%-**^**-**^**9.6%)**	**0.01**
Estrone	1.96	0.66	**−41.1% (-60.5%-**^**-**^**12.0%)**	0.59	**−47.0% (-63.9%-**^**-**^**22.3%)**	**0.001**
Estradiol	2.06	1.36	−41.5% (-65.9%-0.1%)	1.46	−36.0% (-61.8% -7.2%)	0.09
**2-Hydroxylation pathway**	6.41	4.13	−33.6% (-57.2% -3.0%)	5.92	−15.1% (-44.4%-29.6%)	0.48
**2-Pathway catechols**	4.90	3.53	−30.8% (-58.4%-15.1%)	4.18	−20.3% (-51.1%-30.0%)	0.37
2-Hydroxyestrone	4.12	2.42	−31.5% (-59.0%-14.6%)	3.29	−24.9% (-54.3%-23.4%)	0.27
2-Hydroxyestradiol	0.65	0.4	−15.3% (-58.4%-72.6%)	0.80	6.7% (-45.1%-107.2%)	0.83
**2-Pathway methylated catechols**	1.18	0.89	−26.9% (-50.6%-8.1%)	1.39	9.7% (-24.8%-60.0%)	0.54
2-Methoxyestrone	0.72	0.48	−35.8% (-61.3%-6.2%)	0.68	−6.2% (-42.7%-53.4%)	0.85
2-Methoxyestradiol	0.11	0.08	−33.2% (-64.8%-27.0%)	0.12	19.3% (-36.7%-124.8%)	0.60
2-Hydroxyestrone-3-methyl ether	0.2	0.25	−19.4% (-52.8%-37.7%)	0.45	34.9% (-19.1%-124.8%)	0.17
**4-Hydroxylation pathway EM**	1.01	0.67	**−40.1% (-59.2%-**^**-**^**11.8%)**	1.04	−13.6% (-40.6%-25.7%)	0.60
**4-Pathway catechol: 4-Hydroxyestrone**	0.81	0.49	**−39.1%(-61.7%-**^**-**^**3.0%)**	0.73	−18.0% (-47.6%-28.2%)	0.43
**4-Pathway methylated catechols**	0.16	0.13	−14.1% (-49.0%-44.7%)	0.25	48.8% (-10.4%-146.9%)	0.11
4-Methoxyestrone	0.12	0.09	−9.5% (-45.7%-51.0%)	0.15	**69.1% (2.0%-180.4%)**	**0.05**
4-Methoxyestradiol	0.02	0.02	−29.9% (-70.7%-67.7%)	0.07	87.2% (-19.3%-334.2%)	0.08
**16-Hydroxylation pathway**	12.9	15.9	19.5% (-24.8%-90.1%)	13.4	−1.6% (-37.4%-54.8%)	0.95
16α-Hydroxyestrone	1.15	1.22	−6.7% (-42.1%-50.1%)	1.25	−22.1% (-50.6%-22.7%)	0.27
Estriol	5.41	7.24	24.3% (-32.4%-128.6%)	5.85	3.5% (-42.8%-87.6%)	0.91
16-Ketoestradiol	4.28	5.09	20.5% (-22.1%-86.3%)	3.86	−18.5% (-46.8%-25.0%)	0.36
16-Epiestriol	0.88	0.77	−20.6% (-48.9%-23.4%)	0.91	6.4% (-30.5%-63.0%)	0.75
17-Epiestriol	0.15	0.11	−8.8% (-53.5%-79.0%)	0.16	1.6% (-46.2%-91.9%)	0.94
**Ratios**						
**Parent estrogens / estrogen metabolites**	0.27	0.17	**−37.8% (-57.2%-**^**-**^**9.5%)**	0.18	**−32.6% (-53.3%-**^**-**^**2.8%)**	**0.04**
**2-Hydroxylation pathway / parent estrogens**	1.10	1.06	8.7% (-29.4%-67.4%)	1.48	12.0% (-26.4%-70.6%)	0.60
**4-Hydroxylation pathway / parent estrogens**	0.17	0.17	6.0% (-37.4%-79.5%)	0.26	32.5% (-21.2%-122.6%)	0.28
**16-Hydroxylation pathway / parent estrogens**	2.22	4.08	**95.0% (25.5%-202.9%)**	3.34	**57.1% (2.6%-140.5%)**	0.05
**2-Hydroxylation pathway / 16-hydroxylation pathway**	0.50	0.26	**−50.2% (-70.6%-**^**-**^**15.5%)**	0.44	−19.9% (-51.7%-33.0%)	0.41
**4-Hydroxylation pathway / 16-hydroxylation pathway**	0.08	0.04	**−51.4% (-71.0%-**^**-**^**18.6%)**	0.08	−10.1% (-45.7%-48.8%)	0.69
**2-Hydroxylation pathway / 4-hydroxylation pathway**	6.34	6.15	7.6% (-14.9%-36.0%)	5.70	2.6% (-18.9%-29.9%)	0.87
**4-Pathway methylated catechols / 4-pathway catechol**	0.20	0.26	37.3% (-23.3%-145.7%)	0.35	**86.1% (6.6%-225.0%)**	**0.03**
**2-Pathway methylated catechols / 2-pathway catechols**	0.24	0.29	14.4% (-25.4%-75.4%)	0.32	33.6% (-11.2%-100.8%)	0.16

Derived measures of estrogen metabolism and statistically significant estimates are presented in bold font.

^1^ Robust linear regression models were used to estimate percent difference in EM measures with 95% confidence limits across categories of green tea intake while adjusting for age and study center.

^2^ Robust linear regression was used to test for trends in log-transformed EM measures across tea categories. P_trend_ was modeled using categories coded as 0, 1, and 2.

Hypotheses of linear trend across categories of intake were assessed by assigning 0, 1, and 2 to categories and treating the variable as a continuous covariate. Effect modification was considered by stratifying on birthplace (Asia/U.S.), median soy intake, and median BMI. Statistical significance of interactions between tea intake, birthplace, BMI, and soy intake were evaluated by comparing models with and without interaction terms using a likelihood ratio test.

P-values ≤0.05 were considered statistically significant. All tests were two-sided. Analyses were conducted using SAS v. 9.1 (SAS Institute, Cary, NC).

## Results and discussion

In this sample of Japanese American women, median frequency of green tea intake was 1 time per week (interquartile range (IQR): 0.2-7.0). Median frequency of coffee intake was 1 time per day (IQR: 1 time per week-2.5 times per day), while the median frequency of black tea intake was 2-3 times per year (IQR: 0 times per year – 1 times per week).

Table 1 gives characteristics of study participants by menopausal status. Premenopausal participants were younger than their postmenopausal counterparts (with mean ages of 41 and 53, respectively) but had similar, normal BMI (mean 21.7 kg/m^2^ for both groups). Premenopausal participants were more likely than their postmenopausal counterparts to have a history of early menarche (before or at age 12 y, p = 0.04). Daily intake of green tea was more prevalent in postmenopausal than in premenopausal women (33.3% vs. 21.0%, p = 0.049).

In Table 2 we present participant characteristics by frequencies of green tea drinking. Intake of green tea was significantly associated with older age (p = 0.02) and Asian birthplace (p < 0.001). Daily intake of green tea was less prevalent among controls enrolled in Oahu compared to those in Los Angeles and San Francisco (18.1% vs. 37.5% and 43.8%, respectively, p = 0.01). Frequency of green tea intake was also significantly associated with later menarche (*P* <0.001) and higher intake of soy foods (*P* <0.001).

As shown in Table 3, among premenopausal women in luteal phase, intake of green tea was associated with significantly lower urinary concentrations of total estrogens and estrogen metabolites (*P* trend = 0.01). Intake of green tea was significantly associated with lower 16-hydroxylated estrogen metabolites (*P* trend = 0.001).

Among postmenopausal women (Table 4), urinary concentrations of estrone and estradiol declined across categories of green tea intake (*P* trend =0.001 and 0.09, respectively). Each of these was approximately 40% lower in women drinking at least one cup daily compared to those drinking less than one cup per week. Accordingly, the ratio of parent estrogens to all estrogen metabolites decreased across categories of green tea intake (*P* trend =0.04). Finally, the ratio of methylated catechols in the 4-hydroxylation pathway to catechols in the 4-hydroxylation pathway increased significantly (*P* trend =0.03), while the same ratio in the 2-hydroxylation pathway increased non-significantly (*P* trend =0.16).

These associations were apparent both in minimally adjusted analyses and in analyses adjusted for additional potential confounders as described in the methods. Other measures of acculturation and Asian diet, such as Asian birthplace and soy intake, acted as negative confounders of this association, i.e., additional adjustment for these factors strengthened the observed associations between green tea intake and urinary concentrations of parent estrogens. Additional adjustment for caffeine did not change the magnitude or direction of the associations. The estrogens and estrogen metabolites observed to be significantly associated with green tea in premenopausal-luteal and postmenopausal women did not show similar associations with categories of black tea intake (data not shown).

While study findings suggest that green tea intake may influence urinary concentrations of estrogens, the observed associations differed by menopausal status. This may occur because of the marked differences in pre- and postmenopausal women with respect to the levels and sources of systemic estrogens. It is recognized, for example, that tamoxifen and aromatase inhibitors have different efficacies in premenopausal women with intact ovaries, and their postmenopausal counterparts.

In premenopausal women, green tea intake was associated with reduced total estrogens and specifically with markedly reduced 16-pathway EM. No significant association was seen between green tea intake and urinary concentrations of estrone or estradiol. The observed associations did not change in magnitude or direction with adjustment for potential confounders, including measures of acculturation and Asian diet.

In contrast, among postmenopausal women, urinary estrone and estradiol declined significantly across categories of green tea intake. Moreover, among postmenopausal women no trends with green tea intake were seen total EM or for the metabolites of estrone and estradiol, including 16-hydroxylated estrogen metabolites. The associations of green tea with estrone and estradiol observed among postmenopausal women were robust to additional adjustment for measures of acculturation and Asian diet.

The geometric mean urinary concentrations of total estrogens and estrogen metabolites observed in the postmenopausal Japanese-American women from the present study is substantially lower (~50%) than the median urinary levels of total estrogens and estrogen metabolites observed in a recent study of mostly Caucasian postmenopausal women from Western New York [18]. In contrast, mean urinary concentrations of total estrogens and estrogen metabolites measured in premenopausal Japanese-American women during the luteal phase appear to be similar to the mean urinary concentrations observed in Nurses’ Health Study II participants who were in the luteal phase of the menstrual cycle and were mostly Caucasian [19]. Relative representation of different metabolic pathways also appear to differ across these studies, with both pre- and postmenopausal Japanese-American women in the present study showing markedly lower relative concentrations of 2-hydroxylated estrogen metabolites when compared to their respective counterparts [18,19]. It is not clear whether these differences are due to ethnicity, diet or other cultural practices, or technical factors.

Several previous studies have investigated the associations of green tea intake with circulating or urinary estrogens. Consistent with our observation in postmenopausal women, Wu and colleagues [6] observed that in postmenopausal Chinese women in Singapore, green tea intake was associated with lower serum estrone and estradiol. However, Wu and colleagues recently reported on a double-blinded, randomized, placebo-controlled intervention study that was conducted to investigate the effect of a green tea extract containing epigallocatechin gallate (EGCG) on circulating hormones [20]. Postmenopausal women (n = 103) were randomized to receive placebo, 400-mg EGCG, or 800-mg EGCG per day. Serum estrogens and androgens were measured at baseline and after months 1 and 2 of the intervention. Estrogen metabolites were not measured. Investigators did not observe consistent changes in serum estradiol, estrone, or testosterone in any arm of the trial during the 2-month intervention but they did observe statistically significant changes in LDL cholesterol and glucose-related parameters in women given green tea extract. Based upon these findings, the authors suggest that green tea might modify cancer risk through other pathways. It should be considered that differences between the findings of the present study and the randomized feeding trial could reflect differences in the study design or the exposure. While the present study design is more prone than the feeding trial to confounding by unmeasured covariates, it is also possible that a true effect of green tea drinking on estrogen levels is mediated by a component not present in the EGCG isolate used in the trial.

In a previous cross-sectional study of green tea intake among 50 premenopausal Japanese women, Nagata and colleagues found no association of tea with luteal plasma estradiol levels, but did find an inverse association of green tea intake with follicular plasma estradiol concentrations [21,22]. This is consistent with the null association observed between tea and luteal-phase estrogens in the premenopausal women studied here.

The present study is, to our knowledge, the first to measure a broad panel of urinary estrogen metabolites in association with intake of green tea. Findings in premenopausal women could suggest that green tea drinking reduces 16-hydroxylation of estrogens. The inverse association with tea intake was seen for urinary concentrations of nearly all 16-pathway estrogen metabolites. Effects of tea catechins on some CYP450 enzymes with site-selective hydroxylation of parent estrogens (CYP1A and CYP3A) have been observed in laboratory studies, but the net impact of these changes on the *in vivo* estrogen profile is not clear [23,24]. Other possibilities may also be considered. Green tea may affect the half-life of circulating 16-hydroxylated metabolites by modulating their enterohepatic recirculation [25], or, alternatively, their excretion in urine, by modifying activity of the enzymes important in conjugation. A specific effect of green tea on 16-hydroxylation of estrogens should be considered in future studies. In a recent study of urinary estrogens and estrogen metabolites in association with premenopausal breast cancer risk, most 16-hydroxylated estrogen metabolites were not associated with increased risk; however, urinary 17-epiestriol, which in our study shows a significant, inverse trend with green tea intake, was found to be associated with increased risk [26]. To date, the data on this metabolite and its association with breast cancer risk is very limited however, and so the implications of this finding are not clear.

Because aromatization of androgens accounts for most postmenopausal estrogens, lower estrogens in association with green tea intake suggests a possible effect of green tea on aromatase activity. Laboratory-based evidence suggests that green tea catechins may reduce aromatase enzyme activity [23,24,27,28] by modulating expression of CYP19 isoforms [23]. Another study suggests that chronic exposure to tea catechins may be associated with increased expression of this enzyme [29]. The overall impact of exposure to green tea catechins on aromatase-mediated estrogen production is not clear from these laboratory model-based studies. However, mediation of a green tea effect by aromatase is supported by the observation in a previous study, which found that green tea intake and variants in the aromatase gene had multiplicative effects on the risk of endometrial cancer [30].

Observations of increased ratio of methylated catechols to catechols in the 2- and 4-hydroxylation pathways across categories of green tea intake in postmenopausal women could suggest that green tea has an effect on methylation of catechols. In a recent case-control study of breast cancer, the ratio of 4-hydroxylation pathway methylated catechols to 4-hydroxylation pathway catechols was observed to be associated with statistically significantly reduced risk of breast cancer. Catechols in both the 2- and 4- hydroxylation pathways can be oxidized to form quinones; these reactive electrophiles can then react with DNA to form mutagenic adducts [31]. Methylation of the catechols prevents their conversion to reactive quinones and therefore could be expected to be protective with respect to breast cancer risk [32]. Again, it is not clear why this effect would be more apparent among postmenopausal women compared to their premenopausal counterparts.

Estrogens are recognized as important causal factors in the pathogenesis of breast cancer. Prospective studies of postmenopausal women have consistently found increased risk of breast cancer in association with higher circulating [33] and urinary estrogens [34,35]. Recent studies suggest that elevated endogenous estrogens are associated with increased risk of both estrogen receptor positive and negative breast cancers in postmenopausal women [36]. For premenopausal women the association of circulating estrogens with breast cancer risk has not been well supported by the literature, perhaps due to methodologic challenges posed by variability in estrogen levels across the menstrual cycle. Of two studies that carefully accounted for menstrual phase [37,38], one found increased breast cancer risk with higher plasma estradiol during the follicular but not the luteal phase of the menstrual cycle [37] while the other was null [38]. Thus our findings of differences in premenopausal estrogen metabolite profiles by green tea intake have uncertain implications for breast cancer risk . In contrast, our finding that green tea intake is associated with reduced urinary estrone and estradiol in our sample of postmenopausal Japanese-American women, does support the hypothesis that green tea intake may reduce postmenopausal breast cancer risk by modifying exposures to endogenous estrogens.

It should be noted, however, that there are a limited number of studies of green tea intake and risk of breast cancer, and that the evidence from these studies does not consistently support such an association. In particular, of three prospective studies, all conducted in Asia, two had null findings [39,40] while the most recent found reduced premenopausal breast cancer risk in association with regular intake of green tea before the age of 26, and increased breast cancer risk in postmenopausal women with the same exposure [41]. Most studies of green tea have not considered pre- and postmenopausal women separately [42]; only one previous study considered menopausal status as a potential modifier of the association [41]. While adjustment for other measures of acculturation did not lessen the observed associations, it remains possible that green tea intake observed in our study was associated with estrogen profiles as a very sensitive marker of acculturation rather than a causal factor. More prospective studies, with careful assessments of menopausal status and of green tea intake at susceptible times of life, are needed to establish whether green tea is associated with reduced breast cancer risk.

There are a number of strengths of this study worth noting. This population-based sample of Japanese American women recruited in three geographic locations was carefully characterized for varying levels of acculturation. Participants were also queried about breast cancer risk factors and other dietary factors. We used a highly sensitive, specific and reliable assay to measure 15 estrogens and estrogen metabolites in urine [43]. The EM profile is a phenotypic measure and thus provides a direct way to test hypotheses about the effects of dietary and lifestyle factors on estrogen metabolism. Study limitations included the use of questionnaire-based dietary assessment and the lack of information about serving sizes. As is true in any observational study, there is the potential for confounding of associations of tea and EM profiles by unmeasured dietary or lifestyle factors. While we did adjust for regular intake of soy foods, we were not able to adjust for some other dietary factors such as alcohol intake.

## Conclusions

Among postmenopausal Japanese American women, we observed that more frequent intake of green tea was associated with reduced urinary concentrations of estrone. As a rich source of phytochemicals that can interact with and regulate xenobiotic metabolizing enzymes, green tea may modify metabolism [22] or conjugation of estrogens [44] and may thereby impact breast cancer risk. Randomized feeding studies will be helpful to establish the mechanisms by which green tea may modulate cancer risk.

## Competing interests

The authors have declared no conflict of interest.

## Authors’ contributions

AW and RZ participated in the design and coordination of the Asian American Breast Cancer Study from which these participants have been drawn. BF, AW and RZ conceived of the present study. BF and RP performed the statistical analyses and drafted the manuscript. XX, TZ, RZ and LK developed the assay for estrogens and estrogen metabolites and coordinated its first application in the Asian American Breast Cancer Study. XX carried out the assays to measure estrogens and estrogen metabolites in urine samples. All authors read and approved the final manuscript.

## Funding

This work was supported by the Intramural Research Program of the Division of Cancer Epidemiology and Genetics of the National Cancer Institute (NCI), National Institutes of Health (NIH) and contract # HHSN261200800001E to SAIC, Inc. from NCI, NIH, DHHS.
